# Risk–benefit analysis on the clinical significance of convalescent plasma therapy in the management of COVID-19

**DOI:** 10.1136/postgradmedj-2020-138056

**Published:** 2020-08-17

**Authors:** Sivaraman Dhanasekaran, Leela Kagithakara Vajravelu, Venugopal Venkatesalu

**Affiliations:** Department of Pharmacology and Toxicology, Centre for Laboratory Animal Technology and Research, Sathyabama Institute of Science and Technology, Chennai, Tamil Nadu, India; School of Pharmacy, Sathyabama Institute of Science and Technology, Chennai, Tamil Nadu, India; Department of Microbiology, SRM Medical College Hospital and Research Centre, Kancheepuram, Tamil Nadu, India; Department of Internal Medicine, Sundaram Health Centre, Sholinghur, Tamil Nadu, India

The pandemic triggered by the severe acute respiratory syndrome coronavirus 2 (SARS-CoV-2) is already on the list of the greatest threat that human beings have ever encountered in modern history. In the present scenario, SARS-CoV-2 has become a major burden on public health and economic stability of societies around the globe. Currently, there is no known cure attained for treating COVID-19. Epidemic forecast expecting higher incidence of mortality in severely affected zones. Despite global effort in finding suitable vaccine candidates for the pandemic SARS-CoV-2, re-emerging convalescent plasma (CP) therapy is an ideal strategy, which gains paramount importance.^1^ However, this strategy requires some crucial validation with respect to ethical consideration, criteria for donor selection, titer quantification, dose optimisation, limitation factors, chances of occurrence of transfusion events, etc.^2^

CP therapy benefits the society since several years. It has a long history of serving as a viable clinical remedy for treating a range of viral infections such as Spanish influenza, influenza A (H1N1pdm09),^3^ Middle East respiratory syndrome,^4^ Ebola,^5^ SARS^6^ and now for SARS-CoV-2.^7^ Documentary reports suggested that first-ever transfusion of CP attempted during the pandemic spread of Spanish influenza (1918–1920).^8^ In general, CP therapy drives through humoral immunity (antibody-oriented therapy) specific to immunogenic viral antigen. Immunologically individuals with SARS-CoV-2 infection adopt a unique mechanistic pathway in processing antigenic proteins.^9^ Adaptive immune mechanisms substantiate the initial phase of antigen processing, and this often fails in the majority of cases with COVID-19. Clinical outcomes clarify that failure of adaptive immunity in the SARS-CoV-2 infection will call for cytokine storms and thus leads to severe organ dysfunction.^10^ Hence, transfusion of CP in the early possible stage of infection will surely halt the viral replication, thereby flattening the viral load (viremia), and effectively counteracts inflammation-mediated tissue damage.

Efficacy of CP therapy measured in the scale of emerging neutralising antibody titer (NAT) level in the recipient circulation. This implicates the tendency of the NAT to neutralise the viral antigen and lower the viral bioburden.^11^ Results of earlier studies demonstrated that the highest range of seroconversion in SARS-CoV-2 was observed between 8 and 21 days of onset of disease symptoms. Conceptually neutralising antibodies emerge into circulation within 14–21 days of infection. Other findings suggested that higher antibody titer documented in 14 days of mean time point of disease recovery. In this context as per regulatory procedure, the primary level of confirmation on antibody titer will be quantified prior to plasma collection. Outcome of recent clinical investigation on five critically ill cases with COVID-19 reveals that treatment with CP advocates considerable clinical improvement, ensures significant declination in viral load, improves retrieval from ventilation support and demonstrates high level of stability. Further, patients tested negative for viral RNA within 12 days of CP transfusion.^12^ Knowledge gaining part of this study is that quantification index on plasma collection specific for binding titer optimised as 1:1000 IgG and for neutralisation titer >40 dilution offers measurable recovery in treated cases. Another pilot study conducted in 10 cases with COVID-19 subjected to CP therapy reciprocates 100% clinical improvement with titer value >1:640, and 7 of 10 cases turn negative to viral RNA within 7 days of CP (200 mL) transfusion in addition to improved serological and radiology profile.^13^

Recently, the U.S. Food and Dug Administration (USFDA) issued guidelines favouring investigational protocol involving CP therapy for treating patients with COVID-19.^14^ It was observed as a conditional regulation for tier I (clinical trial under Investigational New Drug (IND)), tier II (expanded access for non-trial participants) and tier III (IND emergency therapy); further to it, FDA has not been approved CP regimen for prophylaxis. Informed consent from both donor and recipients has to be obtained prior to the start of the procedure. In case of critically ill recipients, consent may be advocated through attorney or by legally authorised healthcare proxy on their behalf in accordance with healthcare surrogate act.

Two studies currently initiated with a base population of 10–20 patients with COVID-19 for investigating the transfusion benefits of CP as an empirical therapy are in early phase I.^15 16^ Clinical guideline intensifies the following inclusion criteria for donors,: (1) complete resolution of symptoms for at least 14 days prior to donation, (2) completed 28 days of post-recovery period, (3) reveals positive for SARS-CoV-2 antibodies in serology screening, (4) satisfies minimum two negative tests for viral RNA within 48 hours of repetition, (5) possess minimum antibody titer limit (1:160) and (6) donor should be free from other sorts of infections (hepatitis, HIV, syphilis, etc). In some special circumstances, donors are expected to satisfy human leukocyte antigen matching requirements before the start of plasma retrieval. Similarly, recipients should comply with the following inclusion criteria: (1) patients with laboratory-confirmed COVID-19 infection, (2) patients reported as critically ill (severe respiratory failure, septic shock, organ failure) and (3) preferably less than 21 days of report of illness.^17^ Transfusion will be initiated when both donors and recipients satisfy the regulatory needs. Clinical assessment of CP therapy was ascertained based on duration of hospital stay, clinical signs of recovery, change in serology/radiology findings, devoid of ventilation support and mortality index.

In spite of proven clinical efficacy, CP therapy is subjected to certain limitations. The primary limiting factor on availing CP therapy relies on possible transmission of infection from donors, which includes hepatitis B/C, syphilis and HIV.^18^ Hence, adequate measures on screening pre-existing infections and other qualities of donors become highly mandatory. Another potential risk factor in CP therapy is chances of occurrence of life-threatening transfusion events such as anaphylactic shock, circulatory over stack, intensified lung injury, hemolytic reactions.^19^ Potentiation of antibody-dependent enhancement (ADE) is another considerable risk involved in CP transfusion.^20^ ADE occurs due to hyperactive antibodies that emerged in response to earlier viral infection. In cases with COVID-19, these antibodies upon exposure to antigen of different genomic origins further worsen the infection.

In conclusion, globally, there are limited data available on the safety and efficacy of CP in treating patients with COVID-19. Outcome of preliminary studies shows promising therapeutic response; however, large-scale multicentric trials will be needed to ensure the effectiveness of the therapy across the globe.
